# Optical Flow in a Smart Sensor Based on Hybrid Analog-Digital Architecture

**DOI:** 10.3390/s100402975

**Published:** 2010-03-30

**Authors:** Pablo Guzmán, Javier Díaz, Rodrigo Agís, Eduardo Ros

**Affiliations:** Computer Architecture and Technology Department, University of Granada. C/ Periodista Daniel Saucedo Aranda s/n E-18071 Granada, Spain; E-Mails: pguzman@atc.ugr.es (P.G.); jdiaz@atc.ugr.es (J.D.); ragis@atc.ugr.es (R.A.)

**Keywords:** machine vision, intelligent sensors, optical flow, focal plane, motion

## Abstract

The purpose of this study is to develop a motion sensor (delivering optical flow estimations) using a platform that includes the sensor itself, focal plane processing resources, and co-processing resources on a general purpose embedded processor. All this is implemented on a single device as a SoC (System-on-a-Chip). Optical flow is the 2-D projection into the camera plane of the 3-D motion information presented at the world scenario. This motion representation is widespread well-known and applied in the science community to solve a wide variety of problems. Most applications based on motion estimation require work in real-time; hence, this restriction must be taken into account. In this paper, we show an efficient approach to estimate the motion velocity vectors with an architecture based on a focal plane processor combined on-chip with a 32 bits NIOS II processor. Our approach relies on the simplification of the original optical flow model and its efficient implementation in a platform that combines an analog (focal-plane) and digital (NIOS II) processor. The system is fully functional and is organized in different stages where the early processing (focal plane) stage is mainly focus to pre-process the input image stream to reduce the computational cost in the post-processing (NIOS II) stage. We present the employed co-design techniques and analyze this novel architecture. We evaluate the system’s performance and accuracy with respect to the different proposed approaches described in the literature. We also discuss the advantages of the proposed approach as well as the degree of efficiency which can be obtained from the focal plane processing capabilities of the system. The final outcome is a low cost smart sensor for optical flow computation with real-time performance and reduced power consumption that can be used for very diverse application domains.

## 1. Introduction

The term Optical Flow refers to the visual phenomenon due to the apparent movement perceived when we move through a scene and/or regarding the objects moving within it. It represents the projection of the 3-D motion presented in the scene to the 2-D plane of the image sensor or the retina. Note that as a consequence of this protection, depth information is partially lost and the estimation of the 3-D scene structure and motion from the available 2-D field is a very complex task. Optical flow has been extensively studied in the computer vision community (see for instance [1]).

Different approaches have been proposed, in the scientific framework, to estimate the optical flow field. The most widely used ones are the gradient based methods. These methods are based on the constant-brightness assumption. An extended model is the well-known local method proposed by Lucas and Kanade [2]. Another classical model is the one proposed by Horn and Schunck [3], which introduces a global constraint of smoothness to solve the aperture problem. An actual modification suggested by Brox and Bruhn [4] formulates a new approach to solve the Horn and Schunck model’s Achilles heel, the linear smoothness constraint *to satisfy the spatial coherence*; Brox *et al.* propose a non-lineal constraint of smoothness which preserves the optical flow boundaries. Another group of methods are based on local phase correlations. Those methods rely on how the effects of displacement in the spatial domain result in the frequency domain. The use of phase information for optical flow was developed by Fleet and Jepson [5,6]. Correlation techniques are also used in the motion component vector estimation, where block matching methods and similar schemes as the one proposed by Camus [7] are valid alternatives.

In addition to the model choice used to compute the optical flow, its performance and computing resource demands are key elements to develop an embedded system for real-world applications. In the framework of real-time computing approaches, Díaz *et al.* in [8], making use of the Lucas and Kanade [2] approach, developed an embedded system for lane-change decision aid in driving scenarios. Other authors as Mota *et al.* [9] and Köhler [10] propose bio-inspired models based on Reichardt correlators [11] for the design of low cost approaches. In the framework of analog approaches, authors such as Stocker *et al.* [12] present a focal-plane aVLSI sensor to obtain the optical flow components based on the Horn and Schunck model [3] while Mehta and Etienne-Cummings describe a solution based on a normal flow method [13]. Matching techniques are present in the FPGA world where Niitsuma and Maruyama [14] introduce a high performance system able to estimate displacement vectors by means of SAD (Sum of Absolute Differences) matching algorithm.

Following the results of [8,15,16], we focus on Lucas and Kanade’s optical flow method [2], which has been highlighted by the mentioned contributions as a good trade-off between accuracy and performance. In this paper we will focus to obtain a high computational performance (with low accuracy penalty), taking advantage of the analog and digital processors in Eye-RIS™ system to compute optical flow. It is important to remark that this system is a multipurpose machine vision architecture, hence it is not an ad-hoc embedded system to compute optical flow such [10,12–14] which are designed exclusively for this task.

The rest of the paper is organized as follows: Section 2 provides a brief overview of the Eye-RIS™ system which will be the target device to implement the optical flow sensor. Section 3 presents an introduction to the optical flow constraint equation of the Lucas and Kanade method used in this paper. In Section 4, we suggest a number of approaches to enhance the performance of the algorithm implemented in the Eye-RIS™ platform as well as the co-design strategy used to carry out the implementation in this system. The evaluation of the different approaches is described in Section 5. Finally, our experimental results are presented in Section 6 while our conclusions and directions for future research are summarized in Section 7.

## 2. Eye-RIS

In this paper, we make use of a commercial smart camera designed by AnaFocus, named the Eye-RIS™ v1.2 [17] with image resolution of 176 × 144 pixels and capable to operate above 10,000 fps. This Vision System is a compact one which includes all the elements needed for capturing (sensing) images, enhancing sensor operation, and processing the image stream in real-time (as described in this paper), interpreting the information contained in such image flow, and supporting decision-making based on the outcome of such interpretation. Eye-RIS™ system is a multipurpose platform designed to cover the main low-level machine vision primitives with a competitive price in relation with the offered solutions in the market. The present commercial smart cameras provide a reduced collection of machine vision primitives in relation with the Eye-RIS system. On the other hand, we can reuse the same architecture to develop other sort of application much faster that an ad-hoc FPGA solution. Other advantages obtained making use of the focal plane are the GOPS (Giga Operations per Second) and power consumption, where the focal plane [17] (250 GOPS) consume 4mW per GOPS while in a DSP [18] (4.3 GOPS) the obtained consumption is 231 mW per GOPS. Eye-RIS™ system employs an innovative and proven architecture in which image-processing is accomplished following a hierarchical approach with two main levels:

Early-processing: This level comes right after signal acquisition. The basic tasks at this level are meant to extract useful information from the input image stream. Outputs of this level are reduced sets of data comprising image features such as object locations, shapes, edges, *etc.*
Post-processing: Here, the amount of data is significantly smaller. Inputs are abstract entities in many cases, and tasks are meant to output complex decisions and to support action-taking. These tasks may involve complex algorithms within long computational flows and may require greater accuracy than early processing.

One unique characteristic of the Eye-RIS™ vision systems compared to other commercial solutions is that image acquisition and early-processing take place at the sensor, which is actually a Smart Image Sensor (SIS). In this device, image acquisition and pre-processing is performed simultaneously in all pixels of the SIS. Consequently, images do not need to be downloaded from the sensor for the initial stages of the processing. This concept of concurrent sensing-processing extends the Image Sensor concept to the Smart Image Sensor one. The Smart Camera integrates a SIS called Q-Eye, which is a quarter CIF (aka QCIF, 176 × 144) resolution fully-programmable SIS. It consists of an array of 176 × 144 cells plus a surrounding global circuitry. Each cell comprises multi-mode optical sensors, pixel memories, and linear and non-linear analog processors and binary processors. Each cell is interconnected in several ways with its 8 neighboring cells, allowing for highly flexible, programmable, efficient, real-time image acquisition and spatial processing operations. In Smart Image Sensors, each local processor is merged with an optical sensor. This means that each pixel can both sense the corresponding spatial sample of the image and process this data in close interaction and cooperation with other pixels.

Eye-RIS™ v1.2 allows ultra-high processing speed beyond 1,000 frames per second (fps) thanks to the incorporation of mixed-signal processing at the pixel level (enough light is assumed so that exposure time does not become a bottleneck). Processing speed is also application-dependent. Applications with intensive post-processing algorithms might present slower frame rates, since the performance may be constrained by the processing power of the embedded processor (NIOS II).

On the other hand, the Eye-RIS™ Vision System is not conceived for implementing intensive, iterative gray-level processing tasks. This kind of models can be implemented using the embedded micro-processor but its limited computational power highly limits the complexity of the vision models that can be processed in real time. For this reason, it is necessary to take advantage of the resources available in the architecture to develop the proposed approaches in this paper, to estimate optical flow.

The Q-Eye must be seen as a powerful resource for a further processing, *i.e.*, early processing; for this reason, a digital post-processing is needed. Altera NIOS II is a 32-bit RISC digital processor working at 70 MHz clock frequency, integrated in the smart camera, which allows the controlling execution and post-processing image stream provided by the Q-Eye.

The presented architecture has several advantages compared to conventional smart cameras, but imposes some restrictions in programming, due to the analog nature of the SIS Q-Eye, that shall be understood and taken into consideration by application developers.

## 3. Lucas and Kanade Model for Optical Flow Estimation

This section introduces the basics to understand the concept of optical flow and the method used in this paper. An ordered sequence of images allows the apparent motion estimation. The optical flow vector can be defined as a temporal variation in the image coordinates across the time, usually denoted as *v⃑* = (*u*, *v*), and is computed based on the spatio-temporal derivatives of the pixel luminance. To estimate optical flow, a constraint equation is needed. Hence, it typically formulates the constant-brightness hypothesis. The basis of this assumption is that the pixel brightness remains constant over the movement. Thus, we can model this hypothesis with the following expression:

(1) $$\left(\frac{\mathrm{df}(x(t),y(t),t)}{\mathrm{dt}}\right)=0$$

where *f* represents the luminance values of each pixel in the image. Once the hypothesis is defined since (1), is expressed as a derivate of a function with respect to time. Appling the first order Taylor expansion we will obtain the optical flow constraint equation:

(2) $$uf_{x}+vf_{y}+f_{t}=0$$

where *u* and *v* are the optical flow components and the spatio-temporal derivates are represented by *f_x_ f_y_ f_t_* respectively. On the basis of the optical flow constraint equation, Lucas and Kanade [2] proposed the minimization of the error Equation (2) using the sum of the least squares:

(3) $$E(u,v)=\sum_{i\in B}{\left(f_{x}(i)u+f_{y}(i)v+f_{t}(i)\right)}^{2}$$

The objective of minimize the error Equation (3) is to find the displacement components vector *u* and *v*, that minimize the differential error between the previous image warped (making use of the components vector *u* and *v*) and the actual image. Hence the Equation (3) is minimized by partial derivations respect the optical flow vector *v⃑* = (*u*, *v*). The result is presented at Equation (4):

(4) $$\left[\begin{array}{c} u\\ v \end{array}\right]={\left[\begin{array}{cc} \sum_{i\in B}f_{x}^{2}(i) & \sum_{i\in B}f_{x}(i)f_{y}(i)\\ \sum_{i\in B}f_{x}(i)f_{y}(i) & \sum_{i\in B}f_{y}^{2}(i) \end{array}\right]}^{-1}\left[\begin{array}{c} -\sum_{i\in B}f_{x}f_{t}\\ -\sum_{i\in B}f_{y}f_{t} \end{array}\right]$$

where *u* and *v* are the optical flow components, the spatio-temporal derivates are represented by *f_x_ f_y_ f_t_* respectively, and the subscript *i* is the i-th element of the integration block B. Through (4), we estimate the optical flow component vectors from a pair of images of a sequence.

The Lucas and Kanade method has been chosen for two main reasons. At first, this method has been ranked with a very good accuracy *vs.* efficiency trade-off in other literature works [15,16]. As second reason, it is due to the digital processing restrictions in Eye-RIS™ system as explained in section 2 that requires a low complexity model in order to achieve real-time operation.

The next section describes how this model is simplified and optimized (in terms of processing speed) for its implementation in a NIOS II soft-core processor with the focal plane co-processing capability.

## 4. Implementation

One important problem in optical flow methods is the amount of memory accesses and massive multiplications computed by the model. For this reason, a high optimization becomes necessary to obtain a reasonable system performance.

**Figure 1.**
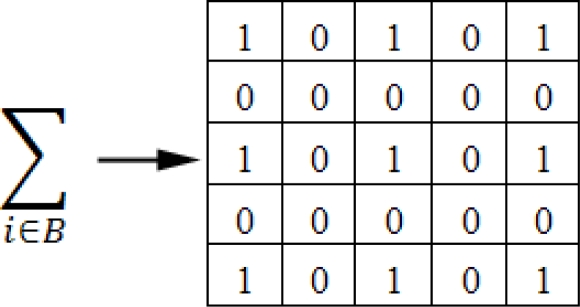
5 × 5 Sparse Integration Block (SIB) representation.

In order to speed up the computation of the Lucas and Kanade model a Sparse Integration Block (SIB) approach is used in (4), as show in Figure 1. Note that each element of the matrix is composed by two image derivatives multiplication (for instance *f_y_* multiply by *f_t_* It) and then sparsely added according the mask values. Each 0 represents missing data and therefore multiplications that are not performed. This translates in high efficiency at reasonable accuracy requiring affordable computational resources. In our model, 9 × 9 and 5 × 5 SIBs are used. In the 9 × 9 SIB case, allows to reduce the computational load from 410 multiplications per pixel to 130 multiplications. This represents an optimization of 68.29% in terms of computational operations and 69.13% in terms of memory accesses with regard to the original one.

**Figure 2.**
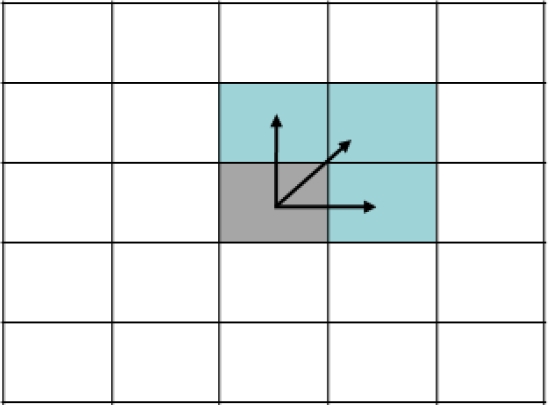
Neighborhood propagation illustration.

We apply the principle of vicinity, which assumes that any point in the image will have a similar value to those in its neighborhood. This principle will be used for the optical flow estimation for computation of only a quarter part of the pixel by 4:1 subsampling. Hence, a calculated optical flow vector will be propagated to the neighborhood as shown in Figure 2 based on the spatial information coherence that says that close pixels tend to have similar optical flow values. This scheme is more accurate than a 4:1 input image sub-sampled pixel grid strategy because the optical flow estimation takes into account the original spatial-temporal derivates in the input images. With this approach, we obtain a factor gain up to four, compared with the original one.

Once the implementation is detailed, we evaluate the system performance with the different approaches. On one hand, the method was implemented in C with two different SIBs; on the other hand, the same implementation was optimized in assembler with different SIBs. Assembler optimization allows to avoid RAW dependencies, optimizes memory accesses in the pipelined data path, and avoids unnecessary stack accesses usually implemented by the C compiler, absolute registers control, loops unrolling, *etc.*

Next, a performance study is carried out to evaluate the optimization evolution. Table 1 shows that the 5 × 5 SIB implemented in assembler reaches a high performance if we compare it with the 9 × 9 SIB versions. Obviously, this is due to the memory access increase as well as to the number of operations.

**Figure 3.**
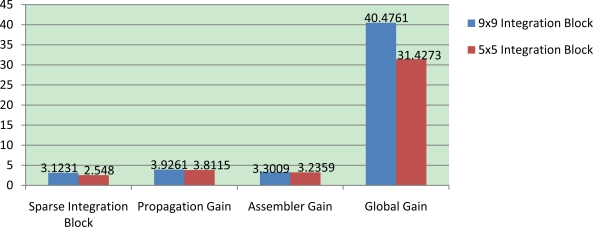
Comparison of the gains obtained with the different optimization strategies. Two different implementations are evaluated here, 5 × 5 (red bars) and 9 × 9 (blue bars). (Left to right) The first group of columns represents the Sparse Integration Block (SIB) factor gain; the second group shows the obtained gain after applying the optical flow 4:1 propagation. In the third column group figure the gain when the method is optimized in assembler while last column show total gain factor obtained after all the approaches are combined.

Therefore, with these approaches, we come to the conclusion that the global gain obtained in a 9 × 9 version amounts to 40.47 the gain factor as show in Figure 3. On the other hand, applying these approaches to the 5 × 5 version, the gain factor is 31.4. The main reason why the 9 × 9 global gain is higher than 5 × 5 optimization is because the 5 × 5 integration stage is significantly reduced (in a factor of 2.7), *i.e.*, the original version uses 25 pixels (in the block) while the SIB version only takes into account 9 pixels; in the meantime the 9 × 9 is reduced to a 3.2, *i.e.*, the original version analyzes 81 pixels while the SIB version analyzes only 25 pixels. Making use of the neighborhood propagation approach, the optical flow is calculated in a quarter of the sequence, which allows the achievement of such high gains. Figure 3 shows the gain factor evolution obtained in each approach and optimization as well as the global gain.

It is convenient extrapolate the performance result to a regular PC processor, for instance the Intel Core 2 Duo, to make clear the constraints we have in the proposed architecture. For a comparative evaluation between NIOS II and an actual processor we make use of Dhrystone 2.1 [19] benchmark. The NIOS II soft-core processor configuration (70 MHz NIOS II/f) used in our test, obtains 71 DMIPS^*2^ while an Intel Core 2 Duo 2.00 GHz processor obtains 4240 DMIPS (using only one of the processor cores). If we compare both processors an Intel Core 2 Duo obtains a gain factor of 59.7. Furthermore, Intel Core 2 Duo uses a superscalar architecture with two cores and support SIMD instructions (MMX and SSE) while NIOS II is a basic processor with a scalar architecture with a reduced instructions set (add, sub, mul, jmp, *etc*.). A study about different optimizations feasible on modern processor is shown in [20]. Therefore, it is important to remark that, using the presented processor, we are much more constrained than using standard ones, and these has motivates to employ the proposed model modifications, analog processor utilization and optimizations techniques.

### 4.1. Pre-Selection of Points of Interest as a Focal Plane Based Attention Mechanism to Speed-Up the Optical Flow Computation

**Figure 4.**
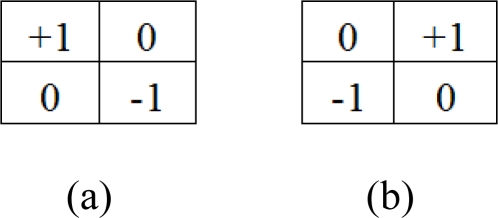
Roberts Cross convolution filter.

The boundaries in an image are areas where optical flow can be more confidently estimated (unless they correspond to 3-D objects where occlusion problems are very common, though this case is less probable). These regions are rich in features; hence, the resulting estimation has more accuracy than in areas with poor contrast structure. This is so because the Lucas and Kanade model collects weighted spatio-temporal structural information. If the local contrast structure is poor, the optical flow estimation confidence will be low. Instead of computing all the points and discarding unreliable points in a second stage, we can avoid the calculations of low confidence optical flow estimates by discarding these points a priori (using local contrast structure estimates). In order to take advantage of this issue, we make use of the Roberts Cross operator to localize the edges (local contrast maxima). The used kernels are shown in Figure 4.

The sum of the absolute value of each convolution provides edge estimations, where each convolution operation obtains the maximum response when the edge angle reaches 45° (Figure 4a) 135° (Figure 4b). The filtering procedure is implemented by applying a Gaussian filter to the edge response and thresholding it with the original signal. The described procedure is indicated in Equation (5):

(5) $$f(x)=\{\begin{array}{l} 1,\mathrm{Gaussian}\;\mathrm{Edge}\;\mathrm{Response}<\mathrm{Edge}\;\mathrm{Response}\\ 0,\mathrm{else} \end{array}$$

**Figure 5.**
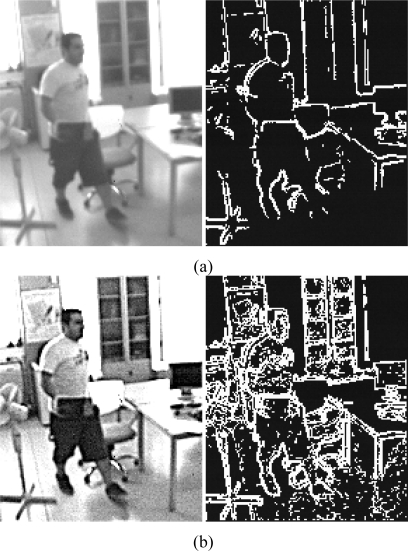
(a) Image edge detection in the original image. (b) Image edge detection with a sharpen pre-filtering.

Low contrast areas will not provide significant edges. This problem can be solved or reduced by locally performing modifications on the image intensity histograms, for instance by applying a 3 × 3 Laplacian convolution (aka Sharpen filter) which emphasizes the low-contrast areas. Figure 5 shows an image edge detection and a sharpen image edge detection.

It can be observed that a higher edge density is obtained by applying the sharpen filter. In this example, Figure 5a has 13.49% of density, while Figure 5b provides a density of 38.20%. Usually, the edge binary map outputs are around 30 up to 40% in high edge density scenes, 50% in the worst case. Using the sharpen filter, this method is more prone to noise. To reduce this noise, we can use the focal plane computational primitives, such as binary dilatation and erosion. Applying successive erosions and dilatations to the binary output includes less noise and becomes sparser before computing the optical flow operations on the NIOS processor. A typical action to remove this noise is applying a 3 × 3 erosion filter followed by a 3 × 3 dilatation filter with a simple filled squared mask as structure element to remove single points.

**Figure 6.**
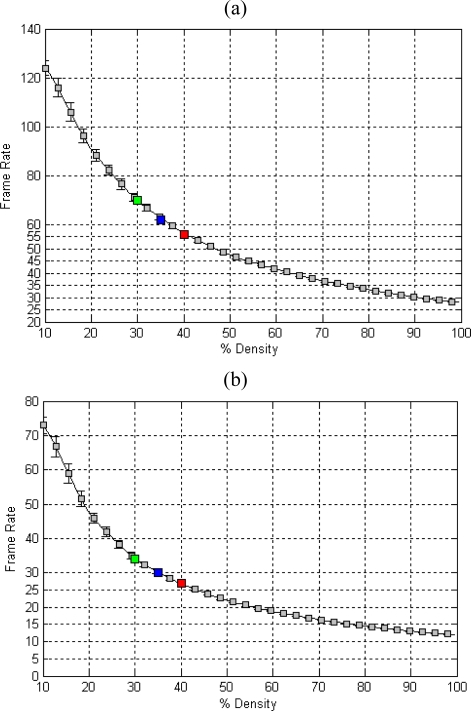
(a) System performance using pre-selected points of interest and 5 × 5 SIB. (b) System performance using pre-selected points of interest and 9 × 9 SIB. Colored mark illustrates three typical scenarios using different image edge densities. The green mark makes reference to the best performance cases (scenes with low edge density), the blue one, to the most common density values (normal scenes) and the red mark, to the worst cases (scenes with high edge density). The error bar represents the standard deviation of the results for 10 trials.

Once we have reached this point, we integrate the edge binary map with the optical flow estimation to optimize the computation time. Since the points of interest are previously selected in the focal plane, this process is carried out on the fly without affecting the global performance. Figure 6 shows the evaluation of this approach. To carry out this experiment we build a synthetic density distribution by filling with 1’s from the beginning until the end of the binary mask, adding a 10% in each step. The first chart, Figure 6a, refers to the 5 × 5 SIB and the second one, Figure 6b, to the 9 × 9 SIB. In this experiment, we measure the frame rate while the edge binary mask density increases. This measurement starts from 10% of density until a density of 100%, *i.e.*, the whole image. In both figures, we remark different scene cases that can be found as well as the standard deviation marked by the error bars.

Analyzing the obtained results and taking into consideration that normal scenes have from 30% to 40% of density with a 35% as mean value, we can deduce that making use of 5 × 5 SIB, the frame rate can oscillate between 56 (red mark in Figure 6a) up to 70 (green mark in Figure 6.a) frames per second with a typical value of 62 fps (blue mark in Figure 6a). In the case of the 9 × 9, the frame rate is around 27 (red mark in Figure 6b) reaching up to 34 (green mark in Figure 6b) frames per second, taking into consideration 30 fps as the usual value (blue mark in Figure 6b).

We can conclude that the proposed approach is suitable for real time computation beyond video-rate, (25 fps). The main advantage of studying optical flow in points of interest (in our case they are pre-selected edges) is the increase of performance. The gain obtained is around 2 in the worse cases, assuming these cases in scenes with a 50% of density. Considering the best cases, scenes with 30% of density, the gain will arise up to 2.6. As commented before, a common scene usually contains 35% of density. Hence, we can conclude that the mean speed up gain is 2.3. This gain can be used to compute flow at higher frame-rate (and therefore, improve the optical flow accuracy [21]) or to include new functionalities into the processors towards final concrete applications (for instance, to implement a tracking system based on the presented approach).

It is important to remark that NIOS II is able to handle 0.044 GOPS to compute optical flow while the focal plane processes 4.1 GOPS to smooth the image, obtain points of interest, and compute the optical flow regularization. To estimate the number of operations used in the focal plane, we carried out an equivalence of a digital processor (NIOS II) to perform the same functionality. Due the amount of operations involved in a previous (focal plane) and final stage (processor) to obtain optical flow, we can conclude that the optical flow estimation could not be implemented in this architecture without the focal plane assistance.

### 4.2. Co-Design Strategy

In previous sections, we defined how to estimate optical flow as well as how such optical flow has been implemented to speed up the optical flow estimations. In this section, we introduce the global description of how to estimate the motion vector components in the Eye-RIS™ system. As described before, the Q-Eye sensor is a system able to process in the same physical layer where the image is captured (focal plane computation). For this reason, at the same time that the system captures the image, we can process it and send it to memory where a post-digital processing takes place.

**Figure 7.**
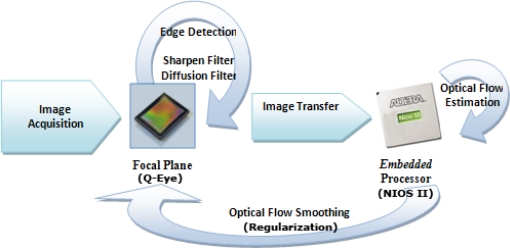
Initial Co-Design scheme.

After the image capture is finished, the focal plane processes the image with the proposed method to select edges, as described in the previous section, and applies a linear diffusion filter [22] that works as smoothing filter of the captured image and improves the numerical computation of the image derivatives. The estimated edge map and the current and previously captured image on the sensor form part of the optical flow method to be computed in the digital processor. Once the optical flow is calculated at the NIOS II processor, a linear diffusion filtering (regularization) is applied to the optical flow components in the focal plane as indicated in Figure 7, to preserve the spatial coherence [3]. Note that when we refer to “regularization”, we mean the process that performs the local averaging process and improves the spatial coherence based on the smoothness constraint.

An important factor to take into account is the exposure time when the image is captured. Adopting a sequential strategy, image acquisition, and NIOS processing being done sequentially (one after the other), is not convenient because it does not take advantage of the pipelined processing capabilities of the system. The focal plane (Q-Eye) is able to work asynchronously with the processor, and the system will be able to capture and process in the focal plane at the same time that we are making use of the digital processor. To accomplish this, the exposure time, focal plane processing, and processor computing time must be taken into account.

**Figure 8.**
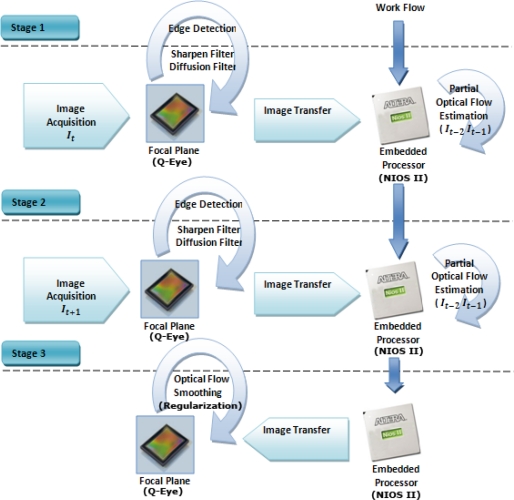
Different stages to estimate optical flow in Eye-RIS™ system.

Furthermore, analog-based internal memories in the focal plane cannot retain the images for a long time due to leakage. Taking into account that the mean value of an image stored in an internal focal plane memory, decreases around 0.8 LSBs every 40 ms. Prolonged storage leads to significant degradation, due to transistors leakage. In order to reduce this signal degradation as well as remain a constant sampling period, we must meet the following constraint, as indicated in expression (6):

(6) $$PT_{\mathrm{Processor}}<\mathrm{ET}+PT_{\mathrm{Focal}\;\mathrm{Plane}}$$

where the time of optic flow processing in NIOS II is *PT_Processor_*, while *ET* is the capture exposure time and *PT_Focal Plane_* is the focal plane processing time. The processing time, in the focal plane, takes approximately 3–40 μs per operation. Hence, we can consider this time to be negligible if we compare it to the exposure time or the processing time on the processor. Although the frame-rate is determined by *PT_Processor_*, if large movements are presented in the scene, we can make use of lower exposure times (to reduce the displacement). In this case, a slightly more complicated scheme is necessary to reduce, as much as possible, the time that each frame is stored in the analog memories. For this purpose, the optical flow process must be split in different stages. In the first stage, the I_t_ image is captured by the focal plane at the same time as the partial optical flow estimation (half of the resolution) of a previous captured sequence I_t−2_ and I_t−1_, is carried out on the processor. The second stage captures I_t+1_ image and processes the unfinished optical flow calculation of the previous stage. The last stage transfers the optical flow components to the focal plane to apply the post-processing lineal diffusion filter which act as a Gaussian isotropic filter. Note that we do not have a continuous acquisition process where time between frames is fixed. Contrary, we handle the acquisition process (according to the scheme of Figure 8) to avoid the image degradation and preserve, as much as possible, the time interval between the captured images. Therefore estimating the flow only between pairs of consecutive frames is possible to avoid the problems previously explained. That is to say, we compute the flow between frames I_t−2_ and I_t−1_ and between frames I_t_ and I_t+1_ but we do not compute the flow between frames I_t−1_ and I_t_ because the time interval can be different. This is because after compute optical flow another further processing algorithms could be applied. Figure 8 illustrates this process.

## 5. Evaluation

The purpose of this section is to evaluate and validate the suggested approaches described in the previous section. In this paper the error measure (7) will be the same that the used by Barron *et al.* [1], which consists in the angular error estimation between the ground-truth optical flow vector and the estimated one:

(7) $$\psi_{E}=\arccos\left({\vec{V}}_{c}\cdot {\vec{V}}_{e}\right)$$

where *ψ_E_* is the estimated error, *V_c_* the true vector flow from the Ground-truth values, *V_e_* the estimated vector flow and → denotes the vector normalization. Note that this error metrics is non linear and combine information from the angular and magnitude error. Nevertheless it is frequently used and therefore has been used for the sake of comparison with other contributions available at the literature.

To evaluate the angular error (7) in a sequence, the real optical flow must be known. The Yosemite sequence, created by Lynn Quam [1], has been widely used for quantitative evaluation of optical flow methods. It is based on an aerial image of the Yosemite Valley, where the ground-truth values of this synthetic sequence are known.

Once the angular error estimation procedure is defined, we carry out an angular error study on the simplified Lucas and Kanade approach described in this work. As a first step, we will evaluate the original model implementation in software, with the different proposed approaches and densities (100% and 48.5%) as shown in Table 2.

To compare our approaches, we will measure the error in the Lucas and Kanade implementation proposed by Barron [1], where the optical flow is estimated making use of a temporal resolution of 5 images and 5 × 5 integration blocks. The table which is shown above indicates that with a 9 × 9 integration block, we obtain better results that working with a 5 × 5 integration block and getting an error similar to the Barron’s implementation. Making use of large blocks, the model weights better the optical flow components but with the associated problem of the computation time. Comparing the angular error between sparse and non-sparse blocks, the table shows that the error is quite similar between them. Hence, by applying sparse integration blocks (SIB), the performance becomes higher regarding the original one.

As we stated in Section 4, the main idea is to propagate the optical flow estimation to the neighborhood. The results obtained above reveal us that making use of this performance optimization; we reach quite similar results if we compare them with non-propagated version. Hence, we can say that this approach is totally valid since the loss in accuracy is insignificant in both sparse and non-sparse integration block approaches.

The last test, we evaluate optical flow regularization (spatial coherence). In this way, we can correct small errors, weighting them with the neighborhood. The most common smoothing filter used is the Gaussian convolution. Hence, we apply the mentioned filter to the estimated optical flow and then, as a priori, the same test scheme is implemented. Analyzing the experiment above, we can deduce that the obtained results are better if we apply this smoothing convolution filter to the optical flow components. The angular error reduction is higher for small integration blocks, both sparse and non-sparse. While using larger masks, the error is lower, if we compare these masks with the smaller ones (5 × 5). This is due to the fact that, for the information collected in small blocks, the model weighted worse than in the case of masks with larger neighborhoods. Hence, applying the regularization to the result helps to weight again the vicinity, being therefore small mask based approaches more favored. If we compare our results, after the regularization step, with the Barron’s implementation we obtain quite similar angular errors but otherwise we reduce the standard deviation by more than a half.

All the previous simplified approaches have been evaluated using Matlab code, with double floating point data representation, to illustrate the effect of the successive approaches. To obtain a realistic evaluation, we will estimate the angular error in the Eye-RIS™ system. In order to carry out these measurements, different integration blocks and post smoothing filters (regularization) are taken into account. In this evaluation, we assume as valid all the approaches and simplifications evaluated before. Note that here a new filter, in the regularization step, is used. This filter, lineal diffusion filter [22], implements a low-pass filter that emulates a Gaussian filter using the Resistive Grid module available on the SIS Q-Eye (focal plane). Due to the nature of the linear diffusion and its equivalence with the Gaussian filter [22], we decided to use this filter because it is more precise and exploits the advantages of the focal plane.

In Table 3 show the measure the average angular error and standard deviation error with different densities (100% and 48.5%).We must remark that the optical flow estimation is developed making use of fix point arithmetic approach.

We conclude that after performing the angular error measurements of optical flow, taking into account different blocks of integration as well as linear diffusion filters (regularization), the best result obtained is the 9 × 9 SIB with a lineal diffusion filter with *σ* = 5.29 as shown in Table 3. The differences with the previous version (Table 2) are mainly produced because we work on fix point arithmetic of 32 bits in NIOS II, properly rescaled across the different processing operations to keep the relevant information. Note that, though bit-width and representation is significantly different than the purely software version (MATLAB version), the results fit quite well the previous data which validate our fixed point implementation.

## 6. Experimental Results

**Figure 9.**
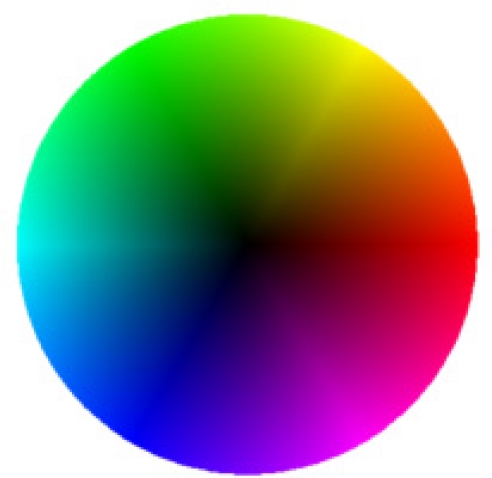
Optical flow representation. The color corresponds with the direction of the optical flow vector while the magnitude is encoded as the color intensity.

In this section, we present our experimental results with real sequences. To evaluate the optical flow results, we have decided to use a traffic sequence where the cars move through the scene. In this sequence, the optical flow has a clear interpretation and therefore, a qualitative evaluation can be done. The original sequence, Ettlinger-Tor, can be obtained from [23]. The optical flow estimation is carried out in different sequences applying both SIBs (5 × 5 and 9 × 9). To interpret the obtained results, the optical flow vector direction is encoded with a color (according to the colored frames of the different images) whereas the vector’s magnitude is expressed by the color intensity as shown in Figure 9.

**Figure 10.**
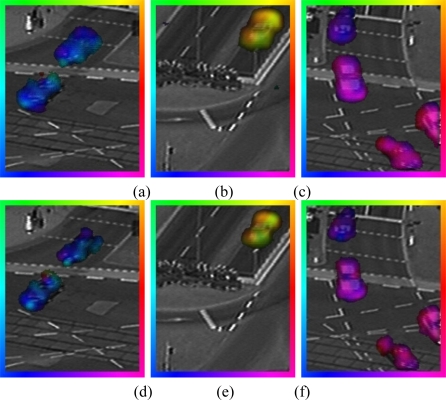
Optical flow estimation in a traffic sequence. Flow field is overlaid with the original frame. In the first Row (a–c), the optical flow is estimated using 9 × 9 SIB. In the second Row (d–f), the optical flow is estimated using 5 × 5 SIB.

In the results shown below we can observe that the optical flow increases, as we increase the sparse integration block (5 × 5 SIB and 9 × 9 SIB). To estimate the optical flow in Figure 10 and Figure 12 we applied to the image a lineal diffusion smoothing, equivalent to a Gaussian filter with σ = 2.5, while the optical flow regularization corresponds to a Gaussian filter with σ = 3.3.

**Figure 11.**
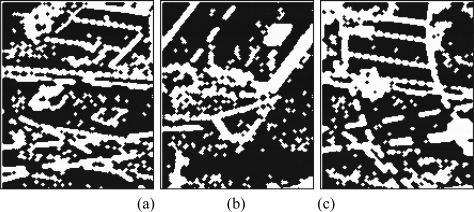
Binary edge map processed, with the proposed method in Section 4.1, in the focal plane.

In Section 4.1, we proposed a method to obtain the image edge response in a focal plane. With this approach, we are able to obtain a mean gain of 2.3. Once the edge estimation is computed in the focal plane, morphological operations of dilatation and erosion are applied to the binary map (two dilatations and one erosion with a 3 × 3 kernel) to bring near the optical flow results to the obtained ones without the sparse estimation. In Figure 11, we show the binary maps processed in the Q-Eye.

**Figure 12.**
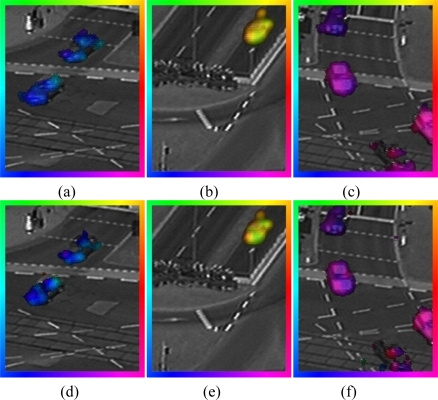
Optical flow estimation, on edges, in a traffic sequence. The average edge density, in these images, is 42.5%. Flow field is overlaid with the original frame. In the first row (a–c), the optical flow is estimated using 9 × 9 SIB. In the second Row (d–f), the optical flow is estimated using 5 × 5 SIB.

After the estimation of the points of interest, the motion vectors are calculated on the processor and post smoothed on the Q-Eye. The results of this procedure are shown in Figure 12. As can be observed, the output density is slightly lower if we compare it with previous results.

We can conclude that using points of interest, the non-edge detection risk in areas of low contrast must be taken into consideration as shown in Figure 12c,f. We are unable to study these areas with the proposed approach. In the case of static cameras, we can also use other pre-selection schemes for reducing the input data stream (instead of local contrast structure), for instance local image change ratio (since most of the scenario will be static). When choosing a method to estimate optical flow, the most appropriate approach depends on the target application and scenario. If the application requires a dense optical flow without the risk of areas of low contrast, a 5 × 5 SIB shall be chosen. When the precision is a crucial factor, pre-selection of point of interest into the estimation of motion vector and using a 9 × 9 SIB is the best choice. If the time is an essential component, the option that best meets these requirements is using 5 × 5 SIB and pre-selection of points of interest to the optical flow estimator.

## 7. Conclusions and Future Research

In this paper, we propose an approach to solve optical flow through the Lucas and Kanade method on hybrid architecture, analog and digital processing, based on a computing focal plane and a digital processor. An early image processing is carried out in the analog device, where the image acquisition and processing are executed in the same physical layer (taking advantage of pixel-wise processing parallelism). Once this early image processing is done, the processor is used to estimate the motion vector components with the different proposed simplifications and optimizations. This co-design strategy allows to improve the input image SNR and at the same time, focuses our attention on the relevant image features. This strategy allows to enhance the system accuracy and performance in terms of computing speed.

In this contribution, we show the different model modifications towards an embedded architecture where computing resources are significantly constrained. The originality and challenge of this work lie in the way that the approach was implemented in this architecture, which has low computation power for digital processing, to obtain reasonable results it is necessary to take full advantage of the powerful capabilities of the analog processor. The presented optical flow implementation, on a platform that integrates analog focal plane processing capabilities and digital processing resources takes full advantage of both computational paradigms. Furthermore, different simplifications and optimizations (such as the post-processing filters) are adapted to better match the computing architecture. The development of vision models in this kind of platforms requires an efficient management of the available processing resources.

Focal plane computations allow pixel-wise processing parallelism. Taking full advantage of these parallelism capabilities is not straightforward and also requires evaluating the signal degradation due to analog processing of storage at the focal plane resources. We have carried out a performance evaluation in terms of processing speed and accuracy as well as the evaluation of different simplifications and optimizations, estimating their impact on the final performance rate and accuracy. The results from the experiment reveal us an empirical validation of the proposed scheme. We can conclude that the obtained implementation (and its performance results) validates the proposed approach, as a high complexity model implemented on a low cost sensor. This article may be useful for those who may have similar restrictions as those exposed here (addressing approaches on hybrid analog-digital platforms) or for those who need to speed-up the models with an affordable loss in accuracy using focal plane analog computing. Future research will focus our work in the automotive sector, to detect car overtaking, where the optical flow is the main factor to carry out these purposes.

## Figures and Tables

**Table 1.** System performance evaluation obtained with a 176 × 144 spatial resolution.

**Integration Block**	**Frame Rate (frames per second)**
L&K C 9 × 9 Integration Block	0.3
L&K C 9 × 9 SIB	0.9
L&K C 9 × 9 SIB	3.6
**L&K ASM 9 × 9 SIB and propagation**	**11.9**
L&K C 5 × 5 Integration Block	1.3
L&K C 5 × 5 SIB	3.3
L&K C 5 × 5 SIB and propagation	8.9
**L&K ASM 5 × 5 SIB and propagation**	**28.8**

**Table 2.** Average angular error (AAE) and standard deviation (STD), in Yosemite sequence (without clouds), with the different proposed approaches and densities on Matlab.

**Integration Block and Used Approach**	**AAE 100%**	**STD 100%**	**AAE 48.5%**	**STD 48.5%**
5 × 5 Barron’s implementation	11.01°	17.14°	10.32°	17.40°
5 × 5	20.68°	21.75°	20.38°	20.57°
9 × 9	14.75°	14.85°	14.63°	14.16°
5 × 5 SIB	19.64°	20.72°	19.34°	19.54°
9 × 9 SIB	14.09°	13.99°	13.98°	13.33°
5 × 5 Propagation	20.74°	21.97°	20.43°	20.84°
9 × 9 Propagation	14.73°	15.04°	14.56°	14.32°
5 × 5 SIB + Propagation	19.71°	20.98°	19.39°	19.86°
9 × 9 SIB + Propagation	14.08°	14.21°	13.92°	13.51°
5 × 5 SIB Propagation + Regularization *σ* = 5.29	12.30°	12.18°	12.10°	11.29°
9 × 9 SIB Propagation + Regularization *σ* = 5.29	10.51°	8.46°	10.51°	8.12°
5 × 5 SIB Propagation + Regularization *σ* = 7.48	11.33°	10.26°	11.17°	9.53°
9 × x9 SIB Propagation + Regularization *σ* = 7.48	9.89°	7.11°	9.86°	6.75°

**Table 3.** Average angular error (AAE) and standard deviation (STD), in Yosemite sequence (without clouds), with the different proposed approaches and densities on Eye-RIS system.

**Integration Block and Used Approach**	**AAE 100%**	**STD 100%**	**AAE 48.5%**	**STD 48.5%**
5 × 5 SIB	24.79°	20.12°	23.45°	18.55°
9 × 9 SIB	17.10°	14.02°	15.88°	12.59°
5 × 5 SIB Propagation	24.84°	20.09°	23.25°	18.41°
9 × 9 SIB Propagation	17.14°	13.88°	15.94°	12.55°
5 × 5 SIB Propagation + Regularization *σ* = 5.29	15.61°	12.01°	13.12°	10.53°
9 × 9 SIB Propagation + Regularization *σ* = 5.29	13.06°	10.11°	11.25°	8.86°
5 × 5 SIB Propagation + Regularization *σ* = 7.48	14.61°	10.63°	12.24°	9.46°
9 × 9 SIB Propagation + Regularization *σ* = 7.48	13.09°	9.34°	10.44°	7.87°
